# Comparison of crestal bone loss and papilla fill after conventional and immediate implant placement: A 12 month clinical and radiographic prospective study

**DOI:** 10.12688/f1000research.131411.1

**Published:** 2023-07-13

**Authors:** Akanksha Raj, Sweta Pradhan, Preetha Shetty, David Kadakampally, Neetha Shetty

**Affiliations:** 1 Ex postgraduate student, Department of Periodontics, Manipal College of Dental Sciences, Mangalore, Manipal Academy of Higher Education, Karnataka, 575001, India; 2 Associate Professor, Gulf Medical University, Ajman, United Arab Emirates; 3 Associate professor, Department of Periodontics, Manipal College of Dental Sciences, Mangalore, Manipal Academy of Higher Education, Karnataka, 575001, India; 4 Professor and HOD, Department of Periodontics, Manipal College of Dental Sciences, Mangalore, Manipal Academy of Higher Education, Karnataka, 575001, India

**Keywords:** Crestal bone, Immediate implants, Conventional Implants, Papillary fill.

## Abstract

Background

The problem of missing teeth persists in all age groups. The main objective of implants in dentistry is to provide a restoration that reconstructs the shape and restores esthetics and functions of edentulous areas. The objectives of this study are to compare the crestal bone level changes and papillary fill after placement of implants in fresh extraction socket, i.e. immediate implant placement, and healed extraction socket, i.e. delayed or conventional implant placement, and to assess other clinical parameters such as modified plaque index (mPI), modified gingival index (mGI) and gingival biotype in between the groups and within the groups.

Methods

18 patients were recruited in the study out of which 9 patients received implants as per immediate implant placement protocol (group 1) and 9 patients received implants as per conventional implant placement protocol (group 2). All patients were evaluated for gingival biotype, mPI and mGI and papillary fill was assessed as per Jemt’s papilla score as clinical parameters. Implant site was assessed for radiographic bone loss using Image J software. Statistical analysis was performed using independent t test, paired t test and chi square test.

Results

At the end of 1 year, results showed that crestal bone loss was seen more in the immediate group than the conventional group. Conventional implants showed better papillary fill than implants placed in fresh extraction sockets. Plaque scores were assessed as per modified plaque index, which showed better results in the conventional group. Modified gingival index was used to assess gingival status which showed better results in the immediate group one year later.

Conclusions

Findings from the study suggest that crestal bone loss was found to be increased in the immediate group than the conventional group and papillary fill was better in the conventional group than the immediate group.

Registration: CTRI ( CTRI/2019/09/021340).

## Introduction

The loss of a single tooth is thought to be a typical esthetic issue that could have psychological effects as well as non-physiological occlusion, that results due to tipping of the neighboring tooth and supra-eruption of the opposing tooth. ^1^^,^^2^ The main objective of implant therapy or modality is to provide a restoration that not only reconstructs the shape but also the esthetics and functions of edentulous areas. Clinical replacement of missing natural teeth with osseointegrated implants has been referred as one of the most notable developments in dentistry. ^3^^,^^4^

Placement of a single tooth implant can either be done in healed extraction socket/site as a conventional or delayed implant, or can be done in a fresh extraction socket/site as an immediate implant procedure. ^5^^,^^6^ Conventionally, placement of a single tooth implant is done in the healed extraction site so as to allow the process of osseointegration to take place which generally takes three to six months. Immediate implants are now being used to achieve optimum esthetic appearance with reduced time of treatment. In this procedure, after creating the implant bed, implants can be placed right away into fresh extraction sockets to establish primary stability. This method is favorable because it helps to preserve alveolar bone, achieves optimum axial positioning of the implant by using the socket as a reference, shortens the edentulous period of three to six months, and requires fewer surgical visits. ^6^^‐^^15^

## Methods

### Ethics and consent

This clinical trial was approved by the Institutional Ethics Committee, Manipal College of Dental Sciences, Mangaluru (ref no. 18120). Initial approval was received on 20 ^th^ October 2018 and finalised on 12 ^th^ November 2018. Participants provided signed informed consent prior to the start of the study. This trial is also registered with CTRI ( CTRI/2019/09/021340). Due to some technical issues with the initial registry, the trial was registered retrospectively. All procedures were conducted in compliance with the Helsinki Declaration 1975.

### Procedure

Study participants who reported to the Department of Periodontology, Manipal College of Dental Sciences, Mangalore with the chief complaint of a missing single tooth were selected as participants in this study. The study was conducted from 1 ^st^ November 2018 to 1 ^st^ December 2020. Before the commencement of the study, the patients were informed of the clinical trial design and were required to sign their informed consent.

### Sample size calculation

$$N=2\;{\left(Z_{1-\alpha /2}+Z_{1-\beta }\right)}^{2}\;\sigma^{2}/d^{2}$$

Using the above formula, the sample size was calculated. Where *Z* (1- *α*/2) = *Z* score for the *α* power chosen. *Z* (1- *β*) = Z score for the power chosen. *σ*1 = standard deviation of group-1

*σ* = average standard deviation. *d* = the minimum difference between in the values with which make clinically relevant impact. With 5% alpha error, 90% power of the study and a clinically significant difference of 0.5 units, the required sample in each group is 9.

### Randomization

The investigator (DK) was in charge of enrolling participants and allocating surgical procedures. The coin toss approach was used for randomization. The lead investigator (AR) performed all surgical operations. A masked investigator (NS) assessed the patients during the recall period and was blinded to all the surgical treatments that had been assigned.

### Inclusion criteria for the patients

1. Age group of 18 to 55 years.
2. Systemically and periodontally healthy individuals.
3. Non-smokers.
4. Good oral hygiene.
5. Availability of follow up.
6. Healthy remaining dentition.
7. Adequate quality and quantity of bone.
8. Retained roots, fractured tooth or tooth advised for extraction due to periapical pathologic lesions.
9. Mandibular/maxillary, anterior/posterior single missing tooth.
10. If buccal cortical plate gets fractured during the procedure of immediate implant placement, the patient will be included in the conventional group.

Patients were excluded from the study if they:

1. Were pregnant at the time of inclusion.
2. Had parafunctional habits.
3. Patients who were not be willing to undergo the treatment.
4. Patients with aggressive periodontitis.

A total of 18 patients with edentulous single tooth were included in the study. Patients of both sexes (7 females and 11 males) in the age range of 18 to 55 years were included in the study. Sex or gender do not impact the bone loss or gain in this study. The study was conducted in a prospective manner and was a comparative clinical trial involving two groups who had a missing single tooth. The patients were those in whom the implants were placed, and prosthesis was yet to be given.

**Figure 1.**
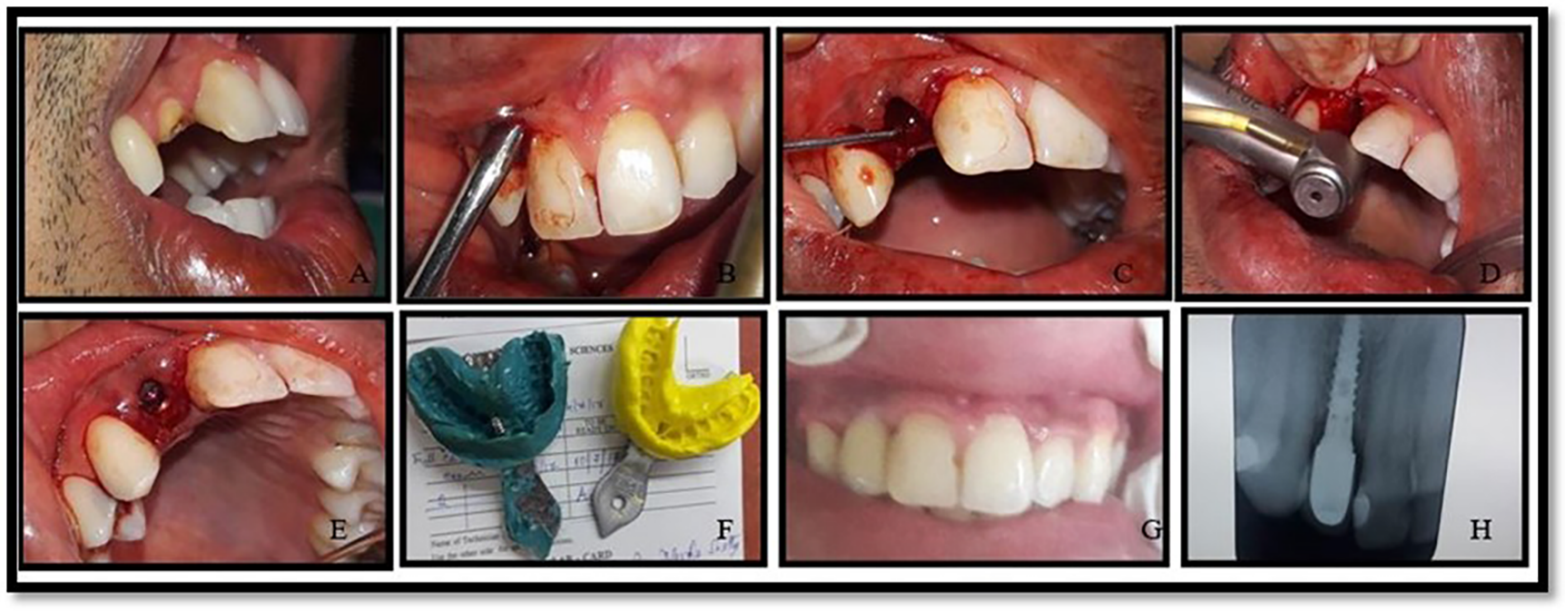
Operative images of implant placement in immediate group (group 1).

**Figure 2.**
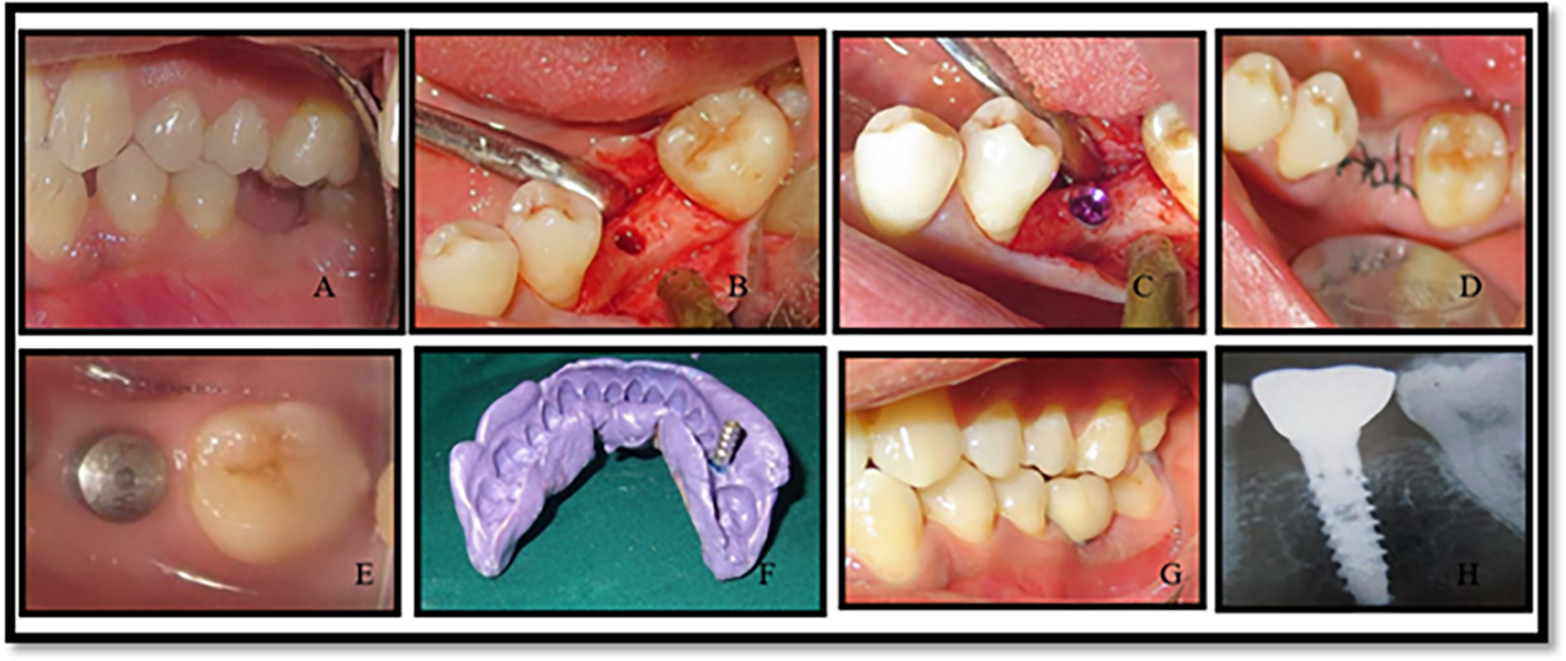
Operative images of implant placments in conventional group (group 2).

**Figure 3.**
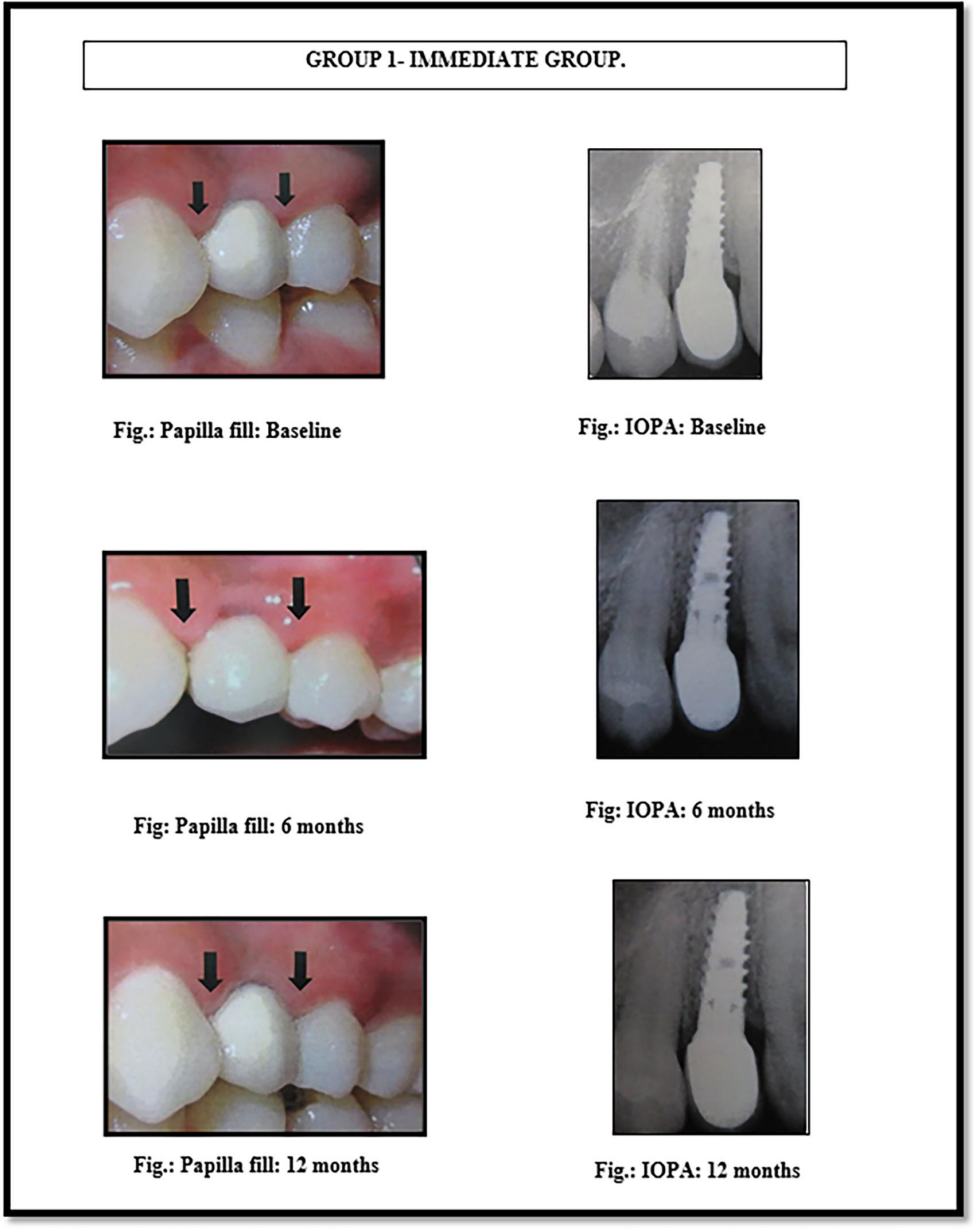
Papillary fill in the immediate implant placement group (group 1) at baseline, 6 months and 12 months.

**Figure 4.**
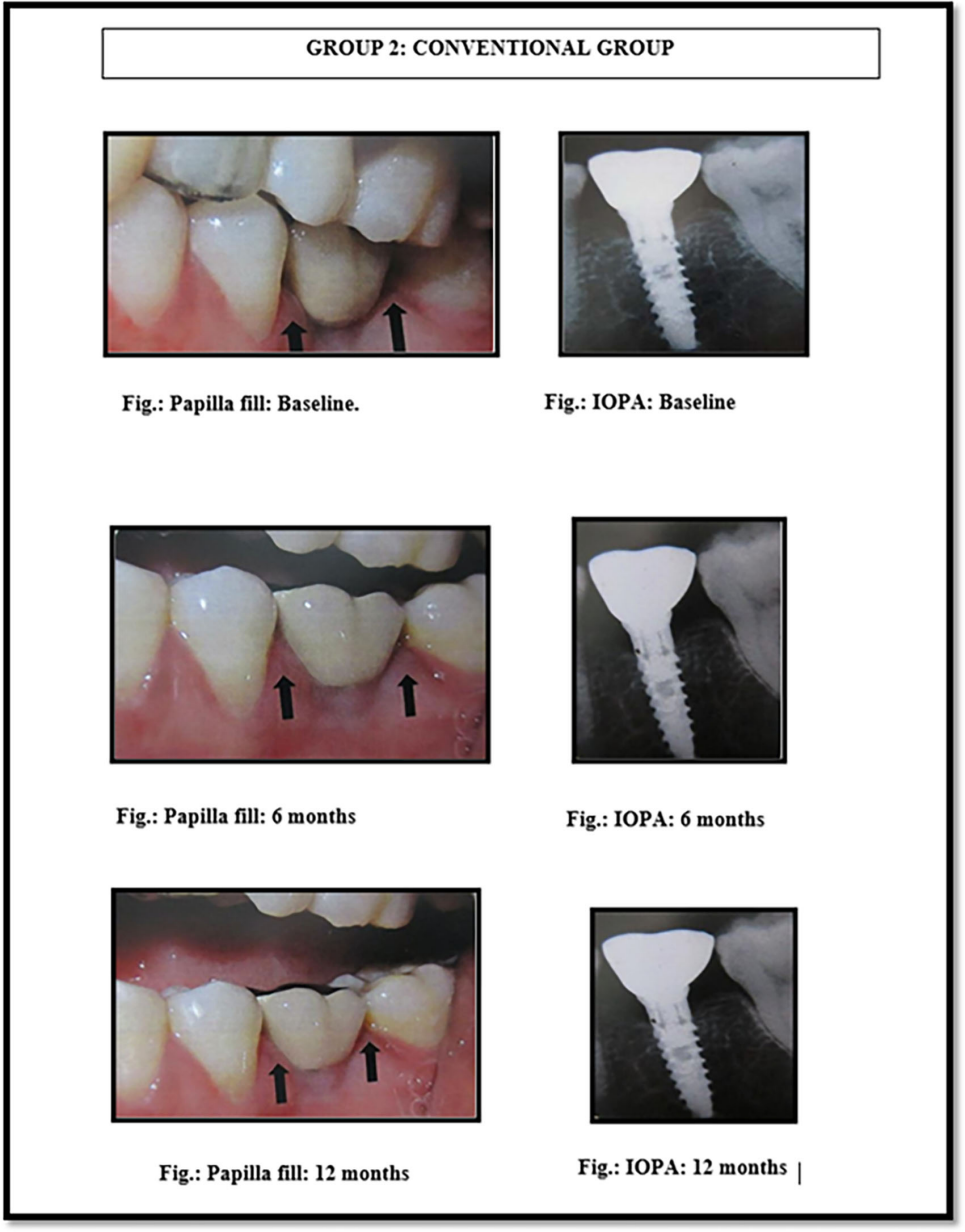
Papillary fill in the conventional implant placement group (group 2) at baseline, 6 months and 12 months.

All the implants used were from the MIS SEVEN implant system (see Figures 1 and 2). The implants were made up of grade V titanium alloy, and of acid etched sand blasted type. Conventional abutments of either cement post or screw retained type were used for the prosthesis. In the 18 patients, 18 implants were placed; of which 12 were placed in the maxilla and 6 were placed in the mandible. All the patients were placed on regular maintenance, with all of them being recalled every 1 month, 6 months and 12 months. At every recall visit, oral hygiene instructions like tooth brushing and use of interdental aids were reinforced. The following clinical parameters were assessed at baseline, 6 months and 12 months after cementation of crown ( Figures 3 and 4): 1. Jemt papilla index (JPI); 2. modified plaque index (mPI); 3. modified gingival index (mGI); 4. gingival biotype.

### Jemt Index (Torsten Jemt, 1997)

This index was used to assess the size of the interproximal papillae adjacent to single implant restorations. ^16^ The assessments were made from a reference line through the highest gingival curvatures of the crown restoration on the buccal side and the adjacent permanent tooth. Index scores were given from 0 to 4 based on the amount of papilla present (see Table 1).

**Table 1.** Jemt Index.

Index/score	Inference
0	Absence of papilla
1	Less than half of the height of the papilla present
2	At least half of the height of the papilla is present and not completely in harmony with adjacent papilla
3	The papilla fills up the entire proximal space and is in good harmony with the adjacent papillae
4	The papillae are hyperplastic and cover much of the single implant restoration and/or the adjacent tooth.

### Modified Plaque Index (Mombelli et al. 1987)

A mouth mirror and William's periodontal probe were used, after air drying of the implant to assess for presence of plaque by running the probe along the margin. The surfaces examined were four gingival areas of the tooth i.e. distofacial, facial, mesiofacial and lingual surfaces.

### Modified Gingival Index (Apse et al. 1991)

A mouth mirror was used to assess the modified gingival index by visual examination on of buccal and palatal/lingual surfaces, without probing.

### Gingival biotype

After administration of local anesthesia, an endodontic file with a rubber stopper was penetrated into the facial soft tissue until it reached the bone. The rubber stopper was placed on the soft tissue surface to mark the position. The thickness of facial soft tissue was equal to the distance between the rubber stopper and the tip of the endodontic file which was recorded by a digital caliper to the nearest 0.1 mm. It is categorized into thick or thin based on the amount of tissue thickness. If it was >1 mm, then it was considered as thick and if it was <1mm, it was considered as thin biotype.

### Radiographic technique and measurements

Intraoral radiographs were taken for all patients at baseline, 6 months and 12 months after crown cementation. A standardized intra oral periapical radiograph (IOPAR) was taken for each selected site using the long cone paralleling technique using a film holder (RINN, XCP, Dentsply Illinois, USA). All radiographs were obtained utilizing an 'E' speed film (Kodak Carestream Health Inc., New York, USA) mount with mm grid scale. These grid scale lines are printed vertically and horizontally at 1mm interval and with bold lines at 5 mm. The factors listed below were taken into account when calculating the degree of bone loss adjacent to the implants. To determine the vertical measures, the fixture abutment junction (FAJ) implant shoulder's horizontal measurement served as a guideline while image analysis software was used for the following radiographic parameters:

1. The most coronal portion of bone that contacts the implant and the vertical distance from the implant's shoulder (FAJ) were given the designation FAJ-I.
2. The most coronal portion of bone facing the neighbouring tooth and the vertical distance from the implant's shoulder were referred to as FAJ-Adj.
3. The soft tissue height was measured from the implant's shoulder to the coronal papilla level.
4. The contact point's vertical distance from the bone's crest was denoted as CP-Bone crest.

All radiographs were scanned using a scanner (HP Scanjet). All radiographic measurements were carried out by using software, Image J. ^17^ Image J (Image processing and analysis in Java) is a pure Java image processing program which can measure area, mean, standard deviation, lengths, angles and min or max of selection or entire image. The Image J analysis was carried out as follows: the image was first transferred to the Image J analysis software, then using the straight-line option, a straight line was drawn to measure 1 mm grid and scale was set by converting distance into pixels. Once the distance was set, all the above-mentioned parameters were measured using the same tool and the amount of bone loss that has occurred was then calculated. For both the groups, the differences in the interproximal crestal bone height between 6 months and 12 months and from baseline and 12 months were calculated. ^18^^‐^^25^

### Statistical analysis

No implant was lost during the observational period. Two patients from the immediate group were lost at 12 months follow up because of COVID-19. Descriptive statistics, mean and standard deviation were calculated for continuous variables. Independent t-test was used to calculate distribution of implants in arches, mean age, crestal bone values i.e., FAJ- I, FAJ-Adj., soft tissue height and CP-BC between the groups. Intragroup comparison was done using paired t-test. Frequency and percentage were calculated for categorical variables. Chi square test was used to calculate intergroup variables in categorical values. Modified plaque index (mPI), modified gingival index (mGI), Jemt's papilla index (mesial & distal) and gingival biotype was calculated using Chi square test. A value of <0.05 is considered to be statistically significant. Microsoft Excel, SPSS version 1.52t and Image J software were used for analysis.

## Results

Crestal bone loss values for group 1 (FAJ - I = 2.5 ± 0.3; FAJ - Adj. = 2.09 ± 0.21; STH = 3.98 ± 1.2 and CP - BC = 6.06 ± 0.99) and for group 2 (FAJ - I = 2.26 ± 0.62; FAJ - Adj. = 1.9 ± 1.03; STH = 4.75 ± 0.32 and CP - BC = 5.94 ± 0.85) at the end of 1 year showed that crestal bone loss was seen more in the immediate group than the conventional group ^58^ ( Tables 2- 5). Papilla scores at the end of 1 year showed that distal papilla fill was better in the immediate group (with score 3 in 14.3% of patients) than the conventional group (with score 3 in 11.1% of patients) and mesially, scores were better in the conventional group (with score 3 in 22.2% of patients) than the immediate group where none of the patients had a score of 3 on the Jemt papilla index. So, conventional implants showed less bone loss and better papillary fill than implants placed in a fresh extraction socket. Plaque scores were assessed as per modified plaque index (mPI) which showed better results in the conventional group (score 1 in 88.9% of patients) than the immediate group (score 1 in 85.7% of patients). Modified gingival index (mGI) was used to assess gingival status which showed better results in the immediate group (100% of patients showed score 1) than the conventional group (88.9% patients showed score 1 and 11.1% of patients showed score 2) at the end of 1 year. Also, gingival biotype did not appear to affect changes in crestal bone level or papilla fill. Sex or gender of the patient does not impact the bone loss or gain in this study.

**Table 2.** The vertical distance from the shoulder of the implant (FAJ) and the most coronal part of bone contacting the implant (FAJ-I) between the groups at baseline, 6 months and 12 months.

FAJ-I	Groups	Mean ± SD	t	p-value
**FAJ–I baseline**	Group 1	2.03 ± 0.36	2.712	0.015 *
	Group 2	1.52 ± 0.43		
**FAJ-I 6 months**	Group 1	2.25 ± 0.28	1.573	0.144
	Group 2	1.89 ± 0.64		
**FAJ-I 12 months**	Group 1	2.5 ± 0.3	1.038	0.319
	Group 2	2.26 ± 0.62		
**Difference between BL - 6 months**	Group 1	13.48 ± 17.78	-1.057	0.306
	Group 2	24.09 ± 24.32		
**Difference between 6m - 12 m**	Group 1	12.42 ± 12.7	-1.071	0.302
	Group 2	22.44 ± 21.96		
**Difference between BL - 12 m**	Group 1	29.38 ± 36.05	-1.206	0.248
	Group 2	51.81 ± 37.57		

* p value is statistically significant.

**Table 3.** Intergroup comparison of FAJ-Adj. - at baseline, 6 months and 12 months.

FAJ-Adj.	Groups	Mean ± SD	t	p value
**FAJ-Adj. baseline**	Group 1	1.6 ± 0.33	0.184	0.858
	Group 2	1.54 ± 0.99		
**FAJ-Adj. 6 months**	Group 1	1.94 ± 0.34	0.44	0.67
	Group 2	1.78 ± 1.04		
**FAJ-Adj. 12 months**	Group 1	2.09 ± 0.21	0.519	0.617
	Group 2	1.9 ± 1.03		
**Difference between BL - 6 months**	Group 1	24.34 ± 29.98	0.299	0.768
	Group 2	20.64 ± 21.82		
**Difference between 6 m - 12 m**	Group 1	6.66 ± 15.66	-0.625	0.542
	Group 2	13.35 ± 24.6		
**Difference between BL - 12 m**	Group 1	36.17 ± 19.67	-0.117	0.909
	Group 2	38.22 ± 42.66		

**Table 4.** Intergroup comparison of soft tissue height at baseline, 6 months and 12 months.

Soft tissue height	Groups	Mean ± SD	t	p value
**Soft tissue height baseline**	Group 1	4.48 ± 0.91	- 1.839	0.085
	Group 2	5.07 ± 0.31		
**Soft tissue height 6 months**	Group 1	4.26 ± 0.87	- 1.963	0.067
	Group 2	4.88 ± 0.34		
**Soft tissue height 12 months**	Group 1	3.98 ± 1.2	- 1.835	0.088
	Group 2	4.75 ± 0.32		
**Difference between BL - 6 months**	Group 1	- 4.89 ± 1.8	-0.859	0.403
	Group 2	-3.77 ± 3.46		
**Difference between 6m - 12 m**	Group 1	- 6.59 ± 8.4	-1.344	0.2
	Group 2	-2.67 ± 2.42		
**Difference between BL - 12 m**	Group 1	-11.66 ± 8.34	-1.623	0.15

**Table 5.** Intergroup comparison of distance between Contact point - Bone crest (CP - BC) at baseline, 6 months and 12 months.

CP - BC	Groups	Mean ± SD	t	p value
**CP - BC baseline**	Group 1	5.91 ± 0.84	2.281	0.037 *
	Group 2	5.13 ± 0.59		
**CP - BC 6 months**	Group 1	6.1 ± 0.98	1.275	0.221
	Group 2	5.62 ± 0.56		
**CP - BC 12 months**	Group 1	6.06 ± 0.99	0.258	0.8
	Group 2	5.94 ± 0.85		
**Difference between BL - 6 months**	Group 1	3.08 ± 5.04	-1.42	0.187
	Group 2	10.67 ± 15.22		
**Difference between 6m - 12 m**	Group 1	2.71 ± 3.15	- 0.651	0.525
	Group 2	5.73 ± 11.88		
**Difference between BL - 12 m**	Group 1	6.13 ± 8.11	-1.619	0.128
	Group 2	16.37 ± 15.03		

* p value is statistically significant.

## Discussion

A crucial indicator for the health of an implant is the quality of the crestal bone. Its preservation is considered an essential indicator for the success of dental implants. Also, the height of the papillae that is related to the interproximal crestal bone contributes to the success of the implant. Thus, papillae preservation height and crestal bone is paramount for fruitful success of the outcome of an implant. Long term studies on the volume of crestal bone loss in the early healing phase, i.e., within one year, generally observe 1.5 mm in the first year, followed by 0.2 mm annually. ^26^ This could be because of occlusal loading forces during mastication and the presence of micro gaps between the implant and placement of the abutment at or apical to the crest, which acts as a plaque retentive area and leads to bone loss. When crestal bone loss occurs, the soft tissue overlying the crestal bone i.e. the papillae would also recede and will lead to plaque accumulation and an unaesthetic appearance. ^27^

In this study, a total of 18 patients were recruited. 9 patients received implants as per immediate implant placement protocol (group 1) and 9 patients received implants as per conventional or delayed implant placement protocol (group 2). Astrand *et al.* ^28^ used two different implant systems (AstraTech and Branemark system) and showed that there was a difference in the pattern of bone remodeling between the implant systems. This can be attributed to the macro and micro characteristics that vary from one system to another. In this study, we have used the MIS SEVEN implant system. It is advantageous to use the same implant system across all patients in the study, as different systems can show different amounts of bone loss. For all the patients, delayed loading protocol was followed, and for all the implants placed, the crown was given after a waiting period of 3-4 months for mandible and 6 months for maxilla. ^29^^,^^30^

The pattern of bone loss after implant placement varies in maxilla and mandible patients, owing to the difference in the density of the bone as it is more cancellous in the maxilla as compared to the mandible. Longitudinal studies have shown a mean difference of 0.23 mm bone loss for implants placed in the maxilla and mandible. ^31^^,^^32^

Excessive surgical trauma and thermal injury may lead to thermal necrosis and fibrous encapsulation of implants. Eriksson and Alberktsson ^33^ reported that the critical temperature for implant site preparation was 47°C for 1 minute or 40°C for 7 minutes. With overheating, the chance of implant failure increases. In this study, adequate internal irrigation was used to keep temperature of bone to minimum. Sharp drills were used, and osteotomy was performed in a staged manner without jumping the drilling sequence.

The height of the bone around the implant has been measured using a variety of techniques. Compared to conventional methods (using magnifying glass), the computerized method is considered to be superior due to higher accuracy. One of these computerized methods is using Image J analysis software, which has high accuracy and precision along with specificity and sensitivity. Grid scale also provides reference distance for the image analysis software so that magnification error is minimized. ^34^^‐^^38^

In this study, all the radiographs were taken at baseline, 6 months and 12 months after crown cementation and were taken using paralleling cone technique with grid overlay and analyzed using Image J analysis. All the radiographs were taken using the same exposure settings which are on the X-ray machine and hence, the standardization was maintained.

The present study showed that values of FAJ-I, FAJ- Adj., CP-BC were higher for group 1 than group 2, indicating that higher crestal bone loss was observed in the immediate group (FAJ-I = 2.5 ± 0.3; FAJ-Adj., = 2.09 ± 0.21; CP-BC = 6.06 ± 0.99) than the conventional group (FAJ-I = 2.26 ± 0.62; FAJ- Adj., = 1.9 ± 1.03; CP-BC = 5.94 ± 0.85) and higher soft tissue height values were seen for group 2 (STH = 4.75 ± 0.32) than group 1 (STH = 3.98 ± 1.2), which shows that the conventional group produced better results than the immediate group. Sasi Kumar *et al.* ^39^ did a study to measure bone loss in conventional and immediate implant placement and stated that bone loss was seen more in the conventional group (1.28 ± 0.24) than the immediate group (1.10 ± 0.39) mm. This is in contrast to the findings in our study; this could be due to the use of autogenous bone chips harvested from the surroundings, which may have aided in better bone formation in immediate group. Tabrizi *et al*. ^40^ demonstrated that the amount of bone loss is significantly ( *P* > 0.05) more in group 2, i.e. conventional (1.6 ± 0.20), than group 1, i.e. immediate, (1.05 ± 0.17) at the 6 month follow up; hence, their results were in contrast to our study. Heinemann *et al*. ^41^ on the other hand concluded that there was a non-significant difference between group 1 and group 2 in approximal bone level change during the first year, which was similar with our study. In a study done by Moustafa *et al.*, ^42^ the immediate loading group showed higher bone loss than the conventional loading group at the end of the 3 year follow up period, which is similar to our findings. Papilla assessment was done immediately on the day of crown cementation, both in maxillary and mandibular dental implants. The crown cementation was done at 3 months in all the patients, thus, standardizing the papillary assessments. The papillary fill around mesial and distal sides of restored dental implants were assessed clinically by scoring papilla from 0 to 4 as per Jemt's papilla index and by digitizing the images at baseline, 6 months and 12 months. In this study, results showed that in the conventional group, papilla scores (mesially) at 12 months showed complete papilla fill in 22.2% of cases and no complete papillary fill was seen in the immediate group. This could be because 2 patients were lost for 12 month follow up from the immediate group due to COVID-19, which is a limitation of our study. Distally, on evaluating Jemt's papilla score, complete papillary fill was seen in 14.3% of patients in the immediate group and 11.1% in the conventional group. These findings were supported by a study done by Grossberg *et al.* (2001), ^43^ in which four patients out of twelve showed complete papilla fill in between the implants. Also, Nariman *et al.* (2018)’s ^44^ study found that both mesially and distally, the conventional group (Mesial side = 1.78 and distal side = 1.89) showed better papilla fill than the immediate group (mesial side = 1.20 and distal side = 1.30) which is in contrast to our study. Lee *et al*. (2005) ^45^ showed that the soft tissue height in between two adjacent implants was 3.3 ± 0.5 mm. This is in accordance with the study done by Rushad Hosi *et al*., ^44^ who also stated that inflammation can lead to false assessment of papillary fill.

Plaque accumulation has been considered as one of the most important causes for crestal bone resorption. ^46^ Formation of plaque on an implant surface is influenced by surface characteristics of the dental implant and abutment material used. ^47^

The patients in the current study were followed at 6 months and 12 months after crown cementation. The modified plaque and modified gingival index scores (mPI and mGI) were used to assess peri-implant mucosal condition at baseline (i.e. on the day of crown cementation), at 6 months and 12 months. In this study, there was no statistically significant mean plaque score difference found between groups at baseline, 6 months and 12 months. This shows that the patients maintained good oral hygiene at 6 months and gradually decreased at follow up time. This is in accordance with Weber HP *et al*. (2000) ^48^ and Renvert S *et al*. (2009). ^49^ Biologic width and biotype are said to influence the peri implant mucosa. In the present study, biotype was divided into two categories: thick and thin. Marginal gingiva often has more effects from gingival biotype than interproximal papillary levels. As in previous investigations, gingival biotype did not appear to affect changes in crestal bone level or papilla fill in this study. ^24^^,^^50^^‐^^57^

A limitation of this study is the smaller sample size. Two patients were lost for 12 months follow up due to COVID-19. The strengths of this study include That the software used in the study to measure bone loss was more accurate than the other graphical methods that had been used in other studies. Additionally, the standardization of implant size and implant system were done in the present study to minimize biasing.

## Conclusion

In consideration of the study's shortcomings, it can be said that:

• Conventionally placed implants showed less bone loss and better papillary fill than implants placed immediately into fresh extraction sockets.
• Gingival biotype had no significant influence with respect to the papilla fill and crestal bone loss irrespective of the technique of implant placement.
• Regardless of the technique of implant placement, there was no bone loss after cementation of crown.

## Patients’ declaration of consent

The authors declare that they have obtained all necessary consent papers from the patients. The patients have signed the form and agreed for their pictures and other clinical information to be published. The names and identities of the patients will remain anonymous.

## Authors’ contributions

All the authors have equal contribution to this research in manuscript preparation, data collection and interpretation.

## Data Availability

**figshare:** Underlying data. https://doi.org/10.6084/m9.figshare.21687209.v1. ^58^ This project contains the underlying following data:
• Data file 1: Master Chart for Immediate implants placement group • Data file 2: Crestal Bone level for immediate implants group • Data file 3: Master Chart for Conventional implants placement group • Data file 4: Crestal Bone level for conventional implants group Data are available under the terms of the Creative Commons Attribution 4.0 International license (CC-BY 4.0). CONSORT checklist and flow diagram for ‘Comparison of crestal bone loss and papilla fill after conventional and immediate implant placement: A 12 month clinical and radiographic prospective study’. https://doi.org/10.6084/m9.figshare.22179419
