# Supplementary material for: Comparison of crestal bone loss and papilla fill after conventional and immediate implant placement: A 12 month clinical and radiographic prospective study
Source: F1000Res. 2024 Jan 8;12:821. Originally published 2023 Jul 13. [Version 2] doi: 10.12688/f1000research.131411.2 (PMC10724648; doi:10.12688/f1000research.131411.2)
Supplement: Peer review history

# Reviewer report: Reviewer response for version 1

**DOI:** 10.5256/f1000research.144252.r225045

**Published:** 2023-12-15

**Authors:** I-Ching Wang

**Affiliations:** 1 The University of Iowa, Iowa City, Iowa, USA

Dear Authors,

 It was suggested that in the results section of the abstract should be more detailed to support the conclusion. Please see the comments below.

**Methods and Material:**

1. Please provide more details of the power calculation including the assumed standard deviation and the effect size. Please clarify if the clinically significant difference in this manuscript was defined as 0.05
2. Please clarify the inclusion criteria for the extraction sockets. Were the defective sockets all included?
3. Please clarify what standardized position on the soft tissue was measured using an endodontic file.
4. Please clarify if a standardized stent for the periapical radiographs was used.

**Conclusions:**

 It should be added the conclusions of observation were at 1 year.

Is the work clearly and accurately presented and does it cite the current literature?

Partly

If applicable, is the statistical analysis and its interpretation appropriate?

I cannot comment. A qualified statistician is required.

Are all the source data underlying the results available to ensure full reproducibility?

No

Is the study design appropriate and is the work technically sound?

Partly

Are the conclusions drawn adequately supported by the results?

Partly

Are sufficient details of methods and analysis provided to allow replication by others?

No

Reviewer Expertise:

Implantology and Periodontology

I confirm that I have read this submission and believe that I have an appropriate level of expertise to confirm that it is of an acceptable scientific standard, however I have significant reservations, as outlined above.

# Reviewer report: Reviewer response for version 1

**DOI:** 10.5256/f1000research.144252.r208643

**Published:** 2023-12-13

**Authors:** Zhen Fan

**Affiliations:** 1 Tongji University, Shanghai, Shanghai, China

The overall research design of the article is reasonable, and the corresponding chapters are also divided into subheadings, which are clear. The following are some suggestions for reference:

 1. The introduction may add more detailed background information to give readers a better understanding of the background and importance of the study.

 2. Objectives of study can be more clearly defined, such as "compare the effects of immediate and delayed implant on the clinical effects of patients (such as long-term bone mass changes, dental health, aesthetic effects, etc.)."

 3. More details can be provided for the method of study, such as the choice of implant type, implant system, and the reason for the surgical methods.

 4. Will the inclusion in the criteria of extraction of teeth caused by periapical diseases affect the effect of immediate implant?

 5. Given that there are many clinical studies on the same subject whose final conclusions are not necessarily consistent, many of the influencing factors (such as the implant system used, the lacking teeth are in the upper or lower jaw, etc.) can analyze the reasons for their inconsistency. This has already been presented in the text, but we suggest a deeper exploration of possible causes and potential clinical significance, rather than just listing the consistent or inconsistent facts. After analyzing these different factors, the need for limiting the conclusions should also be considered.

 6.In the conclusion and discussion, the clinical and practical significance of the corresponding findings could be written and divided more clearly, and we suggest more specific data added to make the results more intuitive.

 7. The research aim in this paper is comparing immediate implant and delayed implant, but one of the studies mentioned in the discussion part is about immediate loading, so it is doubtful whether it is comparable with the research results in this paper.

 8. In this paper, the conclusion of sticking crown and gingival biotype does not affect the treatment results, but according to past studies, soft tissue should have certain influence on bone resorption. More literatures searching and discussion is needed.

 9. In the conclusion part, we can consider further exploring the specific impact of these findings on clinical practice and the future research direction.

 10. There are many ancient documents in the cited documents. Besides some classic documents, it is necessary to add more recent documents related to the research results of this paper, and the ones in recent 3 years are preferred.

Is the work clearly and accurately presented and does it cite the current literature?

Partly

If applicable, is the statistical analysis and its interpretation appropriate?

I cannot comment. A qualified statistician is required.

Are all the source data underlying the results available to ensure full reproducibility?

Partly

Is the study design appropriate and is the work technically sound?

Partly

Are the conclusions drawn adequately supported by the results?

Partly

Are sufficient details of methods and analysis provided to allow replication by others?

Partly

Reviewer Expertise:

dental implantation

I confirm that I have read this submission and believe that I have an appropriate level of expertise to confirm that it is of an acceptable scientific standard, however I have significant reservations, as outlined above.

# Reviewer report: Reviewer response for version 1

**DOI:** 10.5256/f1000research.144252.r194369

**Published:** 2023-10-18

**Authors:** Chandni Batra

**Affiliations:** 1 Indiana University, New Albany, Indiana, USA

The article under review compares crestal bone loss and papilla fill over 12 years after conventional and immediate implant placement. Evaluations included papilla fill or soft tissue height, modified plaque and gingival index, gingival phenotype and radiographic bone loss. The study results demonstrated better papilla fill and crestal bone preservation with no statistical differences between the groups. Overall, the study is well designed and article well written. It would be beneficial to include the following and make following edits:

1. Include the aim of the study and a sentence on what makes this study different from previously completed similar studies in the introduction.
2. Inclusion criteria mentions good oral hygiene - how was this defined? (Plaque score/gingival score?)
3. Inclusion criteria mentions adequate quality/quantity of bone - how was this defined?
4. Terminology gingival biotype can be changed to gingival phenotype as per the 2017 classification system ^1^.
5. Terminology Biologic width can be changed to supracrestal attachment as per 2017 classification system ^1^.
6. Please mention in the results (text) whether the measured differences were significant or not significant for better understanding by readers.

Is the work clearly and accurately presented and does it cite the current literature?

Yes

If applicable, is the statistical analysis and its interpretation appropriate?

Yes

Are all the source data underlying the results available to ensure full reproducibility?

Yes

Is the study design appropriate and is the work technically sound?

Yes

Are the conclusions drawn adequately supported by the results?

Yes

Are sufficient details of methods and analysis provided to allow replication by others?

Yes

Reviewer Expertise:

Periodontology and Implant Dentistry

I confirm that I have read this submission and believe that I have an appropriate level of expertise to confirm that it is of an acceptable scientific standard.

## References

[1] : A new classification scheme for periodontal and peri-implant diseases and conditions - Introduction and key changes from the 1999 classification. *J Clin Periodontol* .2018;45 Suppl 20: 10.1111/jcpe.12935 S1-S8 10.1111/jcpe.12935 29926489
